# Older age groups and country-specific case fatality rates of COVID-19 in Europe, USA and Canada

**DOI:** 10.1007/s15010-020-01538-w

**Published:** 2020-10-24

**Authors:** Christian Hoffmann, Eva Wolf

**Affiliations:** 1 ICH Study Center, 20146 Hamburg, Germany; 2 Department of Medicine II, University of Schleswig-Holstein, Campus Kiel, Kiel, Germany; 3 MUC Research GmbH, 80335 Munich, Germany

**Keywords:** SARS-CoV-2, COVID-19, Case fatality rate, Age distribution

## Abstract

**Purpose:**

To evaluate the association between the percentages of older age groups among confirmed SARS-CoV-2 infections and the country-specific case fatality rate (CFR).

**Methods:**

This ecological study analyzed data from the 20 most severely affected European countries, USA and Canada, in which national health authorities provided data on age distribution and gender among confirmed SARS-CoV-2 cases and deaths.

**Results:**

The proportion of individuals older than 70 years among confirmed SARS-CoV-2 cases differed markedly between the countries, ranging from 4.9 to 40.4%. There was a strong linear association between the proportion of individuals older than 75 years and the country-specific CFRs (*R*^2^ = 0.803 for all countries, *R*^2^ = 0.961 after exclusion of three countries with incongruent data). Each 5% point increase of this older age group among confirmed SARS-CoV-2 cases was associated with an increase in CFR of 2.5% points (95% CI 1.9–3.1).

**Conclusion:**

Data from 20 European countries and the USA and Canada showed that the variance of crude CFR of COVID-19 is predominantly (80–96%) determined by the proportion of older individuals who are diagnosed with SARS-CoV-2. The age distribution of SARS-CoV-2 infections is still far from being homogeneous. Detailed demographic data have to be taken into account in all the analyses on COVID-19-associated mortality. We urgently call for standardized data collection by national health authorities.

**Electronic supplementary material:**

The online version of this article (10.1007/s15010-020-01538-w) contains supplementary material, which is available to authorized users.

## Introduction

In the current pandemic, the country-specific crude case fatality rates (CFRs), the percentage of COVID-19-associated deaths among confirmed SARS-CoV-2 infections, have been the subject of much speculation. Although it became quickly clear that older age is a major risk factor for mortality [1, 2] and that in particular an age of over 70 years is associated with a markedly higher CFR [3, 4], many other factors contributing to regional differences throughout the world have been discussed in recent months.

These factors include not only differences in the overall age structure of the general population of a country and coresidence patterns [5], but also comorbidity burden, obesity prevalence and smoking habits [6] as well as societal and social psychological factors [7]. Others include heterogeneity in testing and reporting approaches [8], variations in health care system capacities and health care [9] and even political regime [10]. Different virus strains [11, 12] or even environmental factors such as air pollution [13–15] have also been discussed, as well as potential differences in genetic variability [16–19] or “trained immunity” induced by certain live vaccines such as bacillus Calmette–Guérin [19–21].

Most articles have considered that older age plays a vital role in influencing severe disease and negative clinical outcomes. However, to clarify the association of older age groups diagnosed with SARS-CoV-2 infection with the country-specific CFR, we have collected available age- and gender-specific data provided from national health authority websites of the 20 most severely affected European countries and of USA/Canada.

## Methods

For Europe, we evaluated the 20 most severely affected larger countries, as defined by numbers per million inhabitants and with at least 1000 confirmed infections. Country-specific confirmed case and death numbers to estimate the country-specific CFR, as well as all available data on age distribution of confirmed cases and deaths were extracted from national health authority websites of European countries and Canada by July 6, 2020 (see supplement). Age-specific data were available for Belgium, Denmark, Germany, Iceland, Ireland, Italy, Moldova, Netherlands, Portugal, Spain, Sweden, Switzerland, and the United Kingdom. For France, only a limited number of cases reported to center laboratories were available. Six countries did not report age-specific data (Armenia, Luxemburg, North Macedonia, Turkey, Serbia and Bosnia Herzegovina) and were replaced by the countries ranking next in order of numbers, namely Austria, Norway, Romania, Estonia, Finland and the Czech Republic. For the USA, we used a detailed report on 1,320,488 laboratory-confirmed COVID-19 cases individually reported to Centers of Disease Control [22].

The numbers of confirmed SARS-CoV-2 cases and deaths were collected as well as the corresponding reported age strata and gender distribution. In countries where only age groups of 65–74 years were available for confirmed cases (see Supplement), we assigned 50% to the group over 70 years. We also performed sensitivity analyses by assigning not only 50% but also 33% and 67% of cases to the younger age groups in countries using different age strata for reporting confirmed SARS-CoV-2 cases (resulting in overlapping strata). For the age group of over 70 years, the percentage of this age group among deaths was assessed. For the association between the country-specific percentage of age group over 70 and age group over 75 years among confirmed infections and the country-specific CFRs, linear regression analyses were performed. Countries were weighed according to their total numbers of COVID-19-associated deaths.

In countries in which age strata and gender distributions were available for both cases and deaths, we estimated the age-stratified CFRs, dividing the absolute death numbers by the cases in a given age group, separately for women and men. Given the heterogeneous reporting in most countries, only three age groups were used, namely < 60 years, 60–79 years and > 80 years. The overall age-specific case fatality risk ratios across countries for men compared to woman (including 95% confidence intervals (CI) and *I*^2^ as measure of consistency/heterogeneity) were estimated using a random effects model applying the method of DerSimonian and Laird. Statistical analyses were performed using Stata/SE 15.1, StataCorp LLC, USA.

## Results

At the time of data-cut (July 6, 2020), a total of about 3.36 million confirmed SARS-CoV-2 cases and 283,792 associated deaths had been documented for the 22 countries, yielding an overall CFR of 7.28%. As shown in Table 1, the country-specific CFR showed a broad spectrum, ranging from 0.6 (Iceland) to 18.1% (France). Six countries reported a CFR of > 10%, namely Belgium, Spain, Italy, Netherlands, France and the United Kingdom. Countries with a relatively low CFR of < 4% were Iceland, Estonia, Moldova, Austria, Norway, Portugal and the Czech Republic.

**Table 1:** Data collected from the national health authority websites (see supplements)

	Cases	Deaths	CFR	Date	Cases with age, %	Deaths with age, %	Cases > 70 years, %	Male cases, %	Male deaths, %	Crude RR, m vs f	Age > 70 years among deaths, %
Austria	18,315	706	3.85	6.7.20	99.99	96.46	16.83	50.00	57.00	1.33	85.46
Belgium	62,016	9766	15.75	6.7.20	98.54	72.25	40.42	37.25	50.43	1.71	85.87
Canada	104,204	8591	8.24	30.6.20	98.88	99.31	24.78	43.88	45.69	1.08	84.40
Czech Rep	12,532	350	2.79	6.7.20	62.82	100	12.02	48.34	57.71	1.46	79.43
Denmark	12,878	607	4.71	6.7.20	100	99.67	17.01	42.76	56.69	1.75	87.44
Estonia	2003	69	3.44	6.7.20	99.75	NA	18.92	44.44	NA	NA	NA
Finland	7257	326	4.49	6.7.20	100	98.16	11.89	49.69	48.00	0.93	88.13
France	164,801	29834	18.10	29.5.20	24.26	NA	35.14	35.02	NA	NA	NA
Germany	196,554	9016	4.59	6.7.20	99.98	100	19.73	48.35	55.35	1.32	85.65
Iceland	1810	10	0.55	6.7.20	99.78	100	4.87	49.72	NA	NA	70.00
Ireland	25,531	1742	6.82	5.7.20	99.85	99.89	21.84	42.82	49.25	1.30	86.41
Italy	241,394	33,900	14.04	23.6.20	99.27	98.94	39.48	45.80	58.06	1.64	85.26
Moldova	17,906	595	3.32	6.7.20	99.78	100	7.84	41.41	51.60	1.51	39.33
Netherlands	50,603	6132	12.12	4.5.20	99.87	100	34.92	37.75	54.99	2.01	88.70
Norway	8930	251	2.81	6.7.20	99.98	100	12.49	49.69	46.61	0.88	87.25
Portugal	44,129	1690	3.83	2.7.20	96.87	95.86	19.65	43.98	50.12	1.28	86.11
Romania	29,620	1799	6.07	11.6.20	96.93	97.55	14.37	45.20	58.97	1.74	52.02
Spain	251,789	28,388	11.27	29.5.20	98.52	72.27	37.23	42.99	56.59	1.73	86.40
Sweden	32,344	1686	7.43	6.7.20	99.98	100	21.10	41.26	54.80	1.73	88.97
Switzerland	73,339	5447	5.21	6.7.20	99.85	100	21.86	46.11	57.47	1.58	89.44
UK	242,764	39,187	16.14	30.6.20	98.53	100	32.66	43.06	56.90	1.75	82.23
USA	1,761,503	103,700	5.89	31.5.20	74.96	68.58	16.63	48.95	54.52	1.25	70.70

*RR* risk ratio, *m vs f* male versus female

The data extent and quality in reporting age- and gender-specific data varied considerably between the countries (supplement). Only a few countries reported different age groups for both cases and deaths stratified by gender. In total, roughly 2.63 million confirmed SARS-CoV-2 cases with available age data could be included in the analysis (82.6% of all reported infections in these countries).

Except for Austria, male patients accounted for less than half of the confirmed diagnoses, with the lowest rates of male patients in France, Belgium and the Netherlands. In contrast, male patients accounted for more than 50% among SARS-CoV-2-associated deaths in most countries. Except for Norway and Finland, in all the countries, male patients had a higher crude mortality risk with a risk ratio varying between 1.08 (Canada) and 2.01 (Netherlands).

The proportions of patients older than 70 years among confirmed SARS-CoV-2 infections differed markedly between countries, ranging from 4.9 (Iceland) to 40.4% (United Kingdom).

As shown in Table 1, the age group older than 70 years accounted for 79–89% of COVID-19-associated deaths in 16 of 20 countries with available data (Estonia and France did not report these rates). In the four countries with lower rates, the proportion of older age groups among deaths increased markedly to 77.1% (Moldova), 78.4% (Romania), 87.5% (USA) and 90% (Iceland) when the age group 60–70 years was included.

**Fig. 1:**
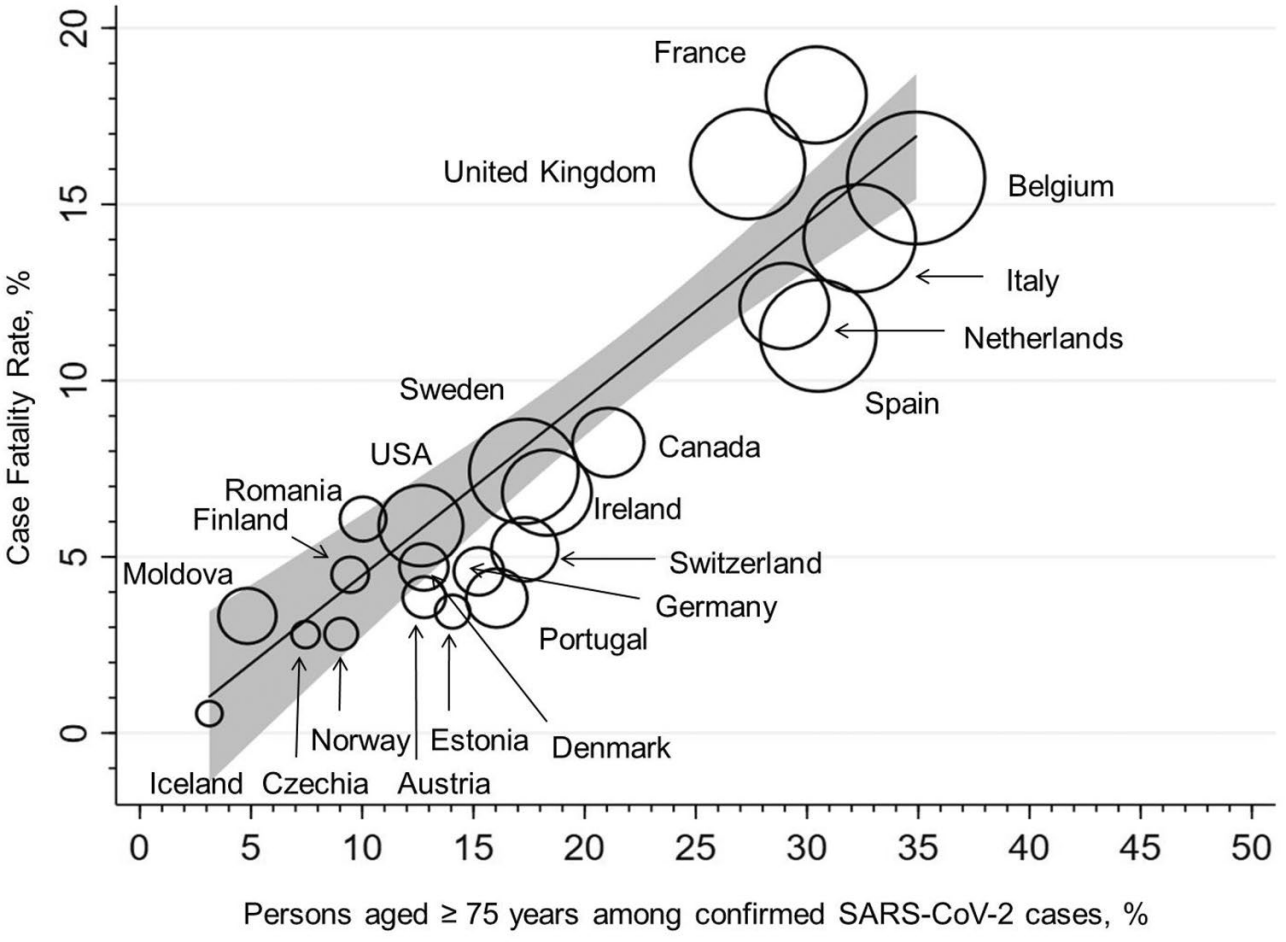
Association between case fatality rate (CFR) and the proportion of persons over 75 years of age among all confirmed SARS-CoV-2 cases (*R*^2^ = 0.8034, *p* < 0.0001). The circle sizes reflect the country-specific numbers of COVID-19-associated deaths per million habitants; the linear fit prediction plot with a 95% confidence interval was estimated by weighted linear regression (weight = total number of COVID-19-associated deaths)

There was a strong association between the proportion of older patients and the country-specific CFRs in our linear regression model (Fig. 1). For the proportion of patients older than 75 years (70 years), *R*^2^ values were 0.803 (0.766), explaining 80.3% (76.6%) of the variance in the country-specific CFR rates. Each 5% point increase of individuals above 75 years among SARS-CoV-2 cases was associated with an increase in overall CFR of 2.5% points (95% CI 1.9–3.1% points). Similar results were seen when adjusting for gender or when an unweighted linear regression was performed (data not shown). Results remained also stable when in countries using different age strata for reporting confirmed SARS-CoV-2 cases (resulting in overlapping strata), 33% or 67% (instead of 50% as in the main analysis) of cases were assigned to the younger age group.

When France (which had a very low rate of infections with known age) and Spain (for which numbers and methodology were corrected several times, implying uncertainty in numbers) were excluded, *R*^2^ values were 0.868 and 0.856 for patients older than 75 and 70 years of age, respectively. If UK is also excluded (as around 66% of the UK data derived from mostly hospitalized patients, probably overestimating CFR), *R*^2^ values were 0.961 and 0.960, respectively.

We have also applied our model to other countries outside Europe that have provided detailed data on age distribution among confirmed SARS-CoV-2 cases such as Australia, Singapore, Japan, China, Republic of Korea or Brazil, yielding very similar results (data not shown).

**Fig. 2:**
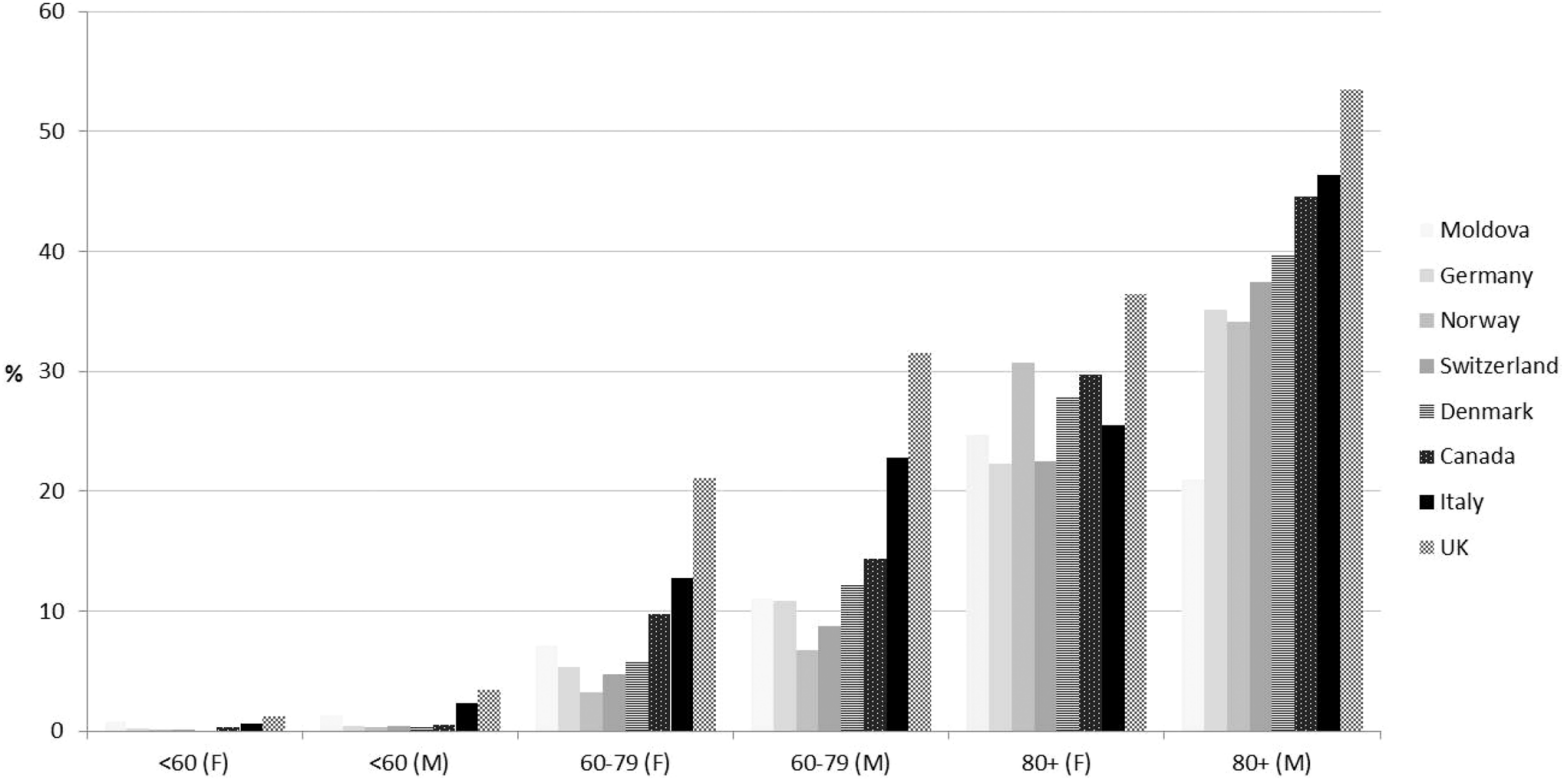
Age- and gender-specific CFR (percentage) for the eight countries in which these data were available. The relative risk (or risk ratio; i.e., RR) for men compared to women was highest in younger age groups. In individuals aged < 60 years, 60–79 years and > 80 years, the estimated overall RR for men versus women was 2.65 (95% CI 2.18–3.21), 1.73 (1.55–1.93) and 1.50 (1.36–1.65), respectively

In Fig. 2, the age- and gender-specific CFR are shown for the eight countries for which these data were available. The UK had the highest CFR in all three age groups, followed by Italy and Canada. Compared to women, CFRs of men were higher in almost all countries across age groups. The estimated overall risk ratio (i.e., relative risk; RR) for men compared to women was highest in younger age groups. In individuals aged < 60 years, 60–79 years and > 80 years, the RR for men was 2.65 (95% CI 2.18–3.21; *I*^2^ = 82%), 1.73 (1.55–1.93; *I*^2^ = 93%) and 1.50 (1.36–1.65; *I*^2^ = 97%), respectively. Of note, country-specific CFRs were all significantly higher for men in the three age groups apart from Moldova and Norway in the age group > 80 years (harboring both a broad confidence interval including a RR of 1).

## Discussion

The striking differences in country-specific case fatality rates observed throughout the world have prompted a huge debate and speculations about the reasons. A myriad of heterogeneous factors contributing to country-specific differences has been evaluated, suggested and proposed during recent months, including genetic, viral, medical, socioeconomic and environmental issues [5–21].

The present analysis of 20 European severely affected countries, USA and Canada strongly indicates that the crude CFR of COVID-19 is predominantly determined by the proportion of patients aged older than 75 years of all individuals diagnosed with SARS-CoV-2 infection. Our simple regression model explained more than 80% of the variance between the countries. Of note, our intention was neither to apply complex mathematical models nor to question the role of other factors contributing to variations in mortality. However, when these factors are discussed and evaluated, detailed information on age distribution among the confirmed SARS-CoV-2 cases have to be taken into account. This is all the more important when countries report relatively low CFRs. In the case of Germany, Russia or India, for example, many explanations have been put forward for the relatively low mortality rates in these countries and have been subject to speculations in the media [8, 23, 24]. Reporting of the age structure of the infected populations is needed before definitive conclusions can be made.

Our data confirm and extend the findings of a recently published early analysis of data reported until April 19, 2020 in which age-standardization of CRFs reduced their variation (standard deviation) by 66% across nine countries [25].

Our data also show that, even in a phase of declining infection numbers (in Europe and Canada), there are still dramatic differences in the proportion of older age groups among confirmed SARS-CoV-2 cases, ranging from 4.9 to 40.4% in European countries. Although we did not have information on testing policies or on different reporting systems of cases and deaths, the present data indicate marked differences between the age distribution of the SARS-CoV-2 epidemic. In many European countries, the distribution of SARS-CoV-2 infections in the general population is still far from homogeneous. We speculate that the marked variation of CFR across countries could diminish over time, especially if less affected countries fail to protect their older age groups. However, the variation of the CFR in specific age groups as shown in Fig. 2 may also decrease with the availability of larger, well-conducted epidemiological studies evaluating the true seroprevalence in countries. We would expect that this would at least diminish the potential bias of regional testing policies, capacities and restrictions for confirmatory testing.

In the countries with available data on gender and age groups in both cases and deaths, we found marked differences between countries and between women and men. In almost all countries, the CFR for men was markedly higher in all age groups, especially in younger age groups. The particularly high rates in the UK may be in part explained by the two-phase official testing programme. Due to limited testing capacity, during the first phase, the strategy was to perform confirmatory SARS-CoV-2 testing only in hospitals and for those with a medical need. Around 66% of the confirmed cases in the UK were derived from the first phase, probably overestimating CFR there.

There is no doubt that our study has several limitations. We could not analyse the age groups more in detail and did not stratify age groups for gender as not many countries reported gender-specific age distribution in both cases and deaths. For some larger countries such as Turkey or France, data on ages were completely missing or incomplete. Many other factors were neglected, such as testing policies and other important health care issues.

However, our simple analysis of 20 European countries and USA and Canada strongly indicates that the crude CFR of COVID-19 appears to be predominantly determined by the proportion of patients older than 70–75 years of age who are diagnosed with SARS-CoV-2. We urgently call for standardized data collection by national health authorities. The numbers of confirmed cases and deaths should be precisely stratified by gender and age groups, preferably grouped at 5-year intervals for all subgroups.

## Electronic supplementary material

Below is the link to the electronic supplementary material.

Supplementary file1 (DOCX 32 kb)

## Data Availability

See supplements.

## Code availability

Statistical analyses were performed using Stata/SE 15.1, StataCorp LLC, USA.
