# Supplementary material for: Older age groups and country-specific case fatality rates of COVID-19 in Europe, USA and Canada
Source: Infection. 2020 Oct 24;49(1):111–6. doi: 10.1007/s15010-020-01538-w (PMC7585357; doi:10.1007/s15010-020-01538-w)
Supplement: Supplementary file 1 — Supplementary file1 (DOCX 32 kb) [file 15010_2020_1538_MOESM1_ESM.docx]

***Supplement Material***

**Table S1**. Presented age strata of SARS-CoV-2 cases and deaths on national health authority websites

|  |  | Confirmed Cases, age strata | | | Confirmed Deaths, age strata | | |
| --- | --- | --- | --- | --- | --- | --- | --- |
|  | Date* | 10-Year  strata (n) | Presented strata** | Gender split | 10-Year  strata (n) | Presented strata** | Gender split |
| Austria | 06.07.20 | yes (10) | 5-4 | No | yes (10) | 5-4 | no |
| Belgium | 06.07.20 | yes (10) | 0-9 | no | yes (6) | 0-9 | no |
| Canada | 30.06.20 | yes (8) | 0-9 | yes | no (5) | other | yes |
| Czechia | 06.07.20 | yes (9) | 5-4 | yes | yes (9) | 5-4 | yes |
| Denmark | 06.07.20 | yes (10) | 0-9 | yes | yes (6) | 0-9 | yes |
| Estonia | 06.07.20 | yes (19) | 0-4 | yes | NA | NA | NA |
| Finland | 06.07.20 | yes (9) | 0-9 | no | yes (5) | 0-9 | no |
| France* | 29.05.20 | yes (5) | other | yes | NA | NA | NA |
| Germany | 06.07.20 | yes (6) | other | yes | yes (11) | 0-9 | yes |
| Iceland | 06.07.20 | yes (9) | 0-9 | no | yes (9) | 0-9 | no |
| Ireland | 06.07.20 | yes (10) | 5-4 | no | yes (10) | 5-4 | yes |
| Italy | 23.06.20 | yes (10) | 0-9 | yes | yes (10) | 0-9 | yes |
| Moldova | 06.07.20 | yes (11) | 0-9 | yes | yes (11) | 0-9 | yes |
| Netherlands | 05.05.20 | yes (20) | 0-4 | no | yes (20) | 0-4 | no |
| Norway | 06.07.20 | yes (10) | 0-9 | yes | yes (10) | 0-9 | yes |
| Portugal | 06.07.20 | yes (9) | 0-9 | no | yes (9) | 0-9 | yes |
| Romania | 05.07.20 | yes (9) | 0-9 | no | yes (9) | 0-9 | no |
| Spain | 04.05.20 | yes (10) | 0-9 | yes | yes (10) | 0-9 | yes |
| Sweden | 06.07.20 | yes (10) | 0-9 | no | yes (10) | 0-9 | no |
| Switzerland | 06.07.20 | yes (9) | 0-9 | yes | yes (9) | 0-9 | yes |
| United Kingdom | 30.06.20 | yes (10) | 0-9 | yes | yes (10) | 0-9 | yes |
| USA | 30.05.20 | yes (9) | 0-9 | yes | yes (9) | 0-9 | yes |

* Date with the last data on age distributions is provided. All websites were assessed on July 6, 2020. **Explanation: 0-9 = 10-19, 20-29, 30-39 etc., 5-4 = 5-14, 15-24, 25-34 etc. 0-4 = 5 yr intervals. Other strata were presented for Canada (20-39, 40-59 etc.), France (15-44, 45-64, 65-74, 75+), and Germany (0-4, 5-14, 15-34, 35-59). For France, only data from central labs were available. NA = not available

Sources:

Austria. Bundesministerium Soziales Gesundheit, Pflege und Konsumentenschutz. Amtliches Dashboard COVID-19. Source: www.info.gesundheitsministerium.at.

Belgium. Sciensano, Belgian institute for health. Covid-19 Belgium Epidemiological Situation. Source https://epistat.wiv-isp.be/covid/covid-19.html

Canada. Public Health Agency of Canada. Public Health Agency of Canada –2020-07-03. Source: https://www.canada.ca/content/dam/phac-aspc/documents/services/diseases/2019-novel-coronavirus-infection/surv-covid19-epi-update-eng-20200703.pdf

Czechia. Ministry of Health of the Czech Republic. COVID‑19: Přehled aktuální situace v ČR. Source: [www.onemocneni-aktualne.mzcr.cz/covid-19](http://www.onemocneni-aktualne.mzcr.cz/covid-19)

Denmark. Sundhedsstyrelsen, Danish Health Authority. Source: https://www.sst.dk/en/English/Corona-eng

Estonia. Health Board, Republic of Estonia. Source: https://koroonakaart.ee/en

Finland. Finnish Institute for Health and Welfare. Source: https://thl.fi/en/web/infectious-diseases-and-vaccinations/what-s-new/coronavirus-covid-19-latest-updates

France. Santé publique France. <https://www.gouvernement.fr/info-coronavirus> and https://www.data.gouv.fr/fr/datasets/donnees-relatives-aux-tests-de-depistage-de-covid-19-realises-en-laboratoire-de-ville/

Germany. Täglicher Lagebericht des RKI zur Coronavirus-Krankheit-2019. Robert-Koch-Institut. Source: rki.de

Iceland. Embætti landlæknis og almannavarnadeild ríkislögreglustjóra standa að baki (The Directorate of Health and The Department of Civil Protection and Emergency Management). Source: https://www.covid.is/english

Ireland. Government of Ireland, Health Protection Surveillance Centre (HPSC) and the Health Service Executive (HSE). Sources: covid19ireland-geohive.hub.arcgis.com and <https://www.hpsc.ie/a-z/respiratory/coronavirus/novelcoronavirus/casesinireland/epidemiologyofcovid-19inireland/COVID-19_Daily_epidemiology_report_(NPHET)_07072020%20v1%20-%20website.pdf>

Italy. Italian Ministry of Health and the Italian Civil Protection. Integrated surveillance of COVID-19 in Italy. Source: https://www.epicentro.iss.it/coronavirus/bollettino/Bollettino-sorveglianza-integrata-COVID-19_23-giugno-2020.pdf

Moldavia. Ministerul Sănătății (Health ministry). Source: http://gismoldova.maps.arcgis.com/apps/opsdashboard/index.html#/d274da857ed345efa66e1fbc959b021b

Netherlands. Rijksinstituut voor Volksgezondheid en Milieu. Epidemiologische situatie COVID-19 in Nederland 5 mei 2020. Source: https://www.rivm.nl/documenten/epidemiologische-situatie-covid-19-in-nederland-4-mei-2020

Norway. Folkehelseinstituttet. Dags- og ukerapporter om koronavirussykdom. Source: https://www.fhi.no/contentassets/ca5914bd0aa14e15a17f8a7d48fa306a/2020_05_05_dagsrapport-norge-covid-19.pdf

Portugal. Ministério da Saúde. SITUAÇÃO EPIDEMIOLÓGICA EMPORTUGAL. Source: covid19.min-saude.pt

Romania. Institutul National de Sanatate Publica. Sources: <https://covid19.geo-spatial.org/statistici/statistici-generale?chart=cazuri-pe-zile> and <https://www.insp.gov.ro/> and http://www.cnscbt.ro/index.php/analiza-cazuri-confirmate-covid19/1854-raport-saptamanal-episaptamana26/file

Spain. Centro de Coordinación de Alertas y Emergencias Sanitarias. Actualización nº 96. Enfermedad por el coronavirus (COVID-19). 05.05.2020(datos consolidados a las 21:00 horas del 04.05.2020) SITUACIÓN EN ESPAÑA. Source: <https://www.mscbs.gob.es/profesionales/saludPublica/ccayes/alertasActual/nCov-China/documentos/Actualizacion_96_COVID-19.pdf>

Sweden. Folkhälsomyndigheten. Sources: <https://experience.arcgis.com/experience/09f821667ce64bf7be6f9f87457ed9aa> and https://www.folkhalsomyndigheten.se/folkhalsorapportering-statistik/

Switzerland. Bundesamt für Gesundheit BAG. Direktionsbereich Öffentliche Gesundheit Situationsbericht zur epidemiologischen Lage in der Schweiz und im Fürstentum Liechtenstein. Source: https://www.bag.admin.ch/bag/de/home/krankheiten/ausbrueche-epidemien-pandemien/aktuelle-ausbrueche-epidemien/novel-cov/situation-schweiz-und-international.html

United Kingdom. Public Health England. Coronavirus (COVID-19) in the UK. Source: [www.coronavirus.data.gov.uk](http://www.coronavirus.data.gov.uk).

USA. U.S. Department of Health and Human Services. Coronavirus Disease 2019 Case Surveillance — United States, January 22–May 30, 2020. Source: https://www.cdc.gov/mmwr/volumes/69/wr/mm6924e2.htm#contribAff
